# Hepatitis C Virus Genetic Variability, Human Immune Response, and Genome Polymorphisms: Which Is the Interplay?

**DOI:** 10.3390/cells8040305

**Published:** 2019-04-03

**Authors:** Daniele Lapa, Anna Rosa Garbuglia, Maria Rosaria Capobianchi, Paola Del Porto

**Affiliations:** 1 Laboratory of Virology, “L. Spallanzani” National Institute for Infectious Diseases, IRCCS, 00149 Rome, Italy; daniele.lapa@inmi.it (D.L.); maria.capobianchi@inmi.it (M.R.C.); 2 Department of Biology and Biotechnology, “C. Darwin” Sapienza University, 00100 Rome, Italy; paola.delporto@uniroma1.it

**Keywords:** hepatitis C virus, viral variability, adaptive immune responses, immune escape, genetics polymorphism, clearance

## Abstract

Hepatitis C virus (HCV) infection is the main cause of chronic hepatitis, affecting an estimated 150 million people worldwide. Initial exposure to HCV is most often followed by chronic hepatitis, with only a minority of individuals spontaneously clearing the virus. The induction of sustained and broadly directed HCV-specific CD4^+^ and CD8^+^ T cell responses, together with neutralizing antibodies (nAb), and specific genetic polymorphism have been associated with spontaneous resolution of the infection. However, due to its high variability, HCV is able to overwhelm the host immune response through the rapid acquisition of mutations in the epitopes targeted by T cells and neutralizing antibodies. In this context, immune-mediated pressure represents the main force in driving HCV evolution. This review summarizes the data on HCV diversity and the current state of knowledge about the contributions of antibodies, T cells, and host genetic polymorphism in driving HCV evolution in vivo.

## 1. Introduction

Hepatitis C virus (HCV) infection is the main cause of chronic hepatitis, affecting an estimated 70 million people worldwide [1]. The long-term consequences of chronic HCV infection include cirrhosis, hepatocellular carcinoma, and end-stage liver disease. Initial exposure to HCV is most often followed by chronic hepatitis, with only a minority (~20%) of individuals spontaneously clearing the virus [2,3]. It is widely assumed that adaptive immune responses play crucial roles in determining the outcome of HCV infection. Indeed, spontaneous elimination of the virus has been associated with the induction of strong and broadly directed virus-specific CD4^+^ and CD8^+^ T cell responses, which are sustained through HCV clearance and beyond [4,5]. In addition, there is an accumulating evidence that neutralizing antibodies (nAb) can contribute to viral clearance [6,7]. One of the mechanisms employed by HCV to evade the adaptive immune response is the generation of large numbers of different, but closely related, viral variants. Indeed, due to the high error rate of the RNA-dependent RNA polymerase and to the high replication rate, HCV circulates in vivo as a complex population, commonly referred as a quasispecies [8]. In this context, adaptive immune responses favour the selection of persistent variant viruses, which encode altered epitopes that are less efficiently or not recognized by T cells or antibodies. Moreover, over the last 10 years, several studies have emphasized the roles of host genetic factors, i.e., favourable genotypes or alleles that could represent a strong indicator of clearance or may be associated to a higher chance of sustained virological response (SVR) in treated patients [9]. In this review, we summarize the current state of knowledge about the contribution of T cells and antibodies in driving HCV evolution in vivo and of host genetic determinants in influencing the course of HCV infection.

## 2. HCV and Quasispecies

**Figure 1:**
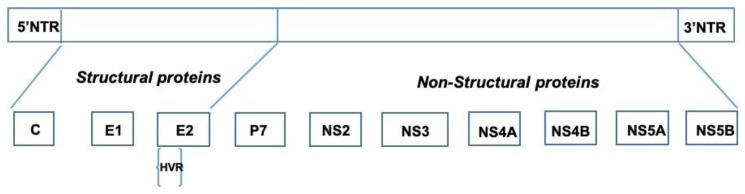
Diagrammatic representation of the 9.6 kb positive stranded RNA genome (top panel) and structural and non-structural proteins (lower panel).

HCV is an enveloped positive-sense RNA virus of the *Hepacivirus* genus in the Flaviviridae family. Its genome of 9600 nucleotides (nt) encodes a polyprotein of about 3000 amino acids. It is processed by host cell peptidases and viral proteases generating three structural (C, or core) and the envelope glycoproteins, 1 and 2 (E1, E2), and seven non structural proteins (p7, NS2, NS3, NS4A, NS4B, NS5A, NS5B) (Figure 1). P7 is a 63 amino acid hydrophobic polypeptide, which is essential for in vivo production of infectious particles, and NS2 has been implicated in viral assembly, envelopment, maturation, and release [10,11,12]. NS3 is a 70 kDa multifunctional protein containing a serine protease. NS3 protease cleaves both viral and host proteins and has been implicated in viral mechanisms for evading innate immune responses. NS4A is a 54 amino acid protein, the shortest non-structural protein, which has a number of functions in viral replication, host immune response modulation, and virus assembly [13]. NS4B is involved in the formation of functional replication complexes, in the assembly of virions particles, and in inhibiting the innate immune activation [14,15,16,17,18]. NS5A influences the replicative capacity of the virus [19]. It also includes the interferon sensitivity determining region (ISDR), where several amino-acid substitutions can influence the efficacy of interferon (IFN) therapy especially in genotype 1b infected patients [20].

NS5B is a 591 amino acid 86 kDa protein, which is cleaved by the NS3 serine protease [21]. The N terminal 530 amino acid portion harbours a subdomain motif that is seen universally in RNA dependent RNA polymerases and requires both magnesium and manganese as co-factors. There is a significant pangenotypic conservation of NS5B, which has allowed the development of efficacious NS5B inhibitors, such as SOFOSBUVIR, which is effective towards all HCV genotypes [22]. The 5′ untranslated region (5′UTR) and 3′ untranslated region (3′UTR) are highly conserved, and they play key roles in the regulation of viral translation.

HCV has three hypervariable regions (HVR) and one of these, HVR1, is considered as the major target of the adaptive immune response, in fact it contains targets both of B and T cell epitopes. HVR1 is a 27 amino-acid region located at the N-terminal of the E2 protein. It has three functional microdomains. The first one, including the amino acid residues, 14, 15, and 25–27, is essential for binding the E2 protein to the scavenger receptor class B, type 1. The second one, encompassing residues at positions 1–13, is dispensable for HCV entry, but it can affect HCV infectivity, whereas the third microdomain (amino acids 14–24) is not relevant for cell entry and it is an epitope for neutralizing antibody (nAb) [23]. HVR2 is a segment of 7 to 11 amino acids and it is required for binding CD81, which is a tetraspanin receptor required for HCV cell entry, but it does not represent a target for immune surveillance [24]. HVR3 is located between HVR1 and HVR2, it includes 17 to 36 amino acids and is involved in the HCV binding process [25,26]. 

HCV is classified into 7 genotypes (GT) and 67 subtypes [27]. This virus displays a high genetic variability due to the high viral replication level and to the RNA-dependent RNA polymerases that lack a proofreading mechanism [28]. More specifically, its mutation rate has been estimated to be in the order from 1.5 × 10^−3^ to 2.0 × 10^−3^ nucleotide substitutions per site per genome per year [29,30,31]. As a result of error-prone polymerase, the viral population circulates in the host as a complex and continuously changing mutant spectrum or mutant cloud known as a quasispecies [32]. The concept of quasispecies is applied to gain a better understanding of the complexity of the viral population and its impact on both virus evolution and resistance to antiviral therapy.

What does the term “quasispecies” exactly mean?

The theory of quasispecies, introduced by Professor Manfred Eigen in 1979, was soon applied to RNA viruses. In this theory, Eigen defined the concept of quasispecies as a group of self-replicating molecules that were different, but closely related, evolving as a single unit when facing changes in the environment [33]. Consequently, due to the impossibility of defining a viral genome as a single sequence, it is rather considered the “weighted average of individual sequences” [34]. In the quasispecies virus theory, the classical dichotomy between mutants and wild type as a match between two separate categories has been rejected. Instead, all of the related variants, regardless of their frequency of representation, are considered replicons, which are characterized by dynamic genetic interactions. After an environmental change, the pre-existing wide range of variants in the original population and the continuous generation of new variants during replication will lead to a rapid selection of the most favourable mutants for new environments. 

In viraemia, the use of the term, “quasispecies”, is justified in vivo because it conveys the concept of an ensemble of similar genome sequences generated by a mutation-selection process. The term “positive selection” indicates situations in which a genotype becomes dominant in an evolving population. The fixed viral population is enriched in individual viruses that display relatively higher fitness values, underscoring the influence of relative fitness. In this context, fitness signifies the replicative capacity of a specific variant under a specific condition (antiviral drug administration, immune pressure), and for this reason, fitness is environment-dependent.

In contrast with positive selection, negative selection is the process by which a variant is eliminated in an evolving population as the result of negative properties that hamper adaptation to the environment. Negative selection frequently causes the elimination of subsets of genomes, but it can also result in their maintenance at low frequencies. These low-frequencies genomes may be detectable or not, depending on the analytical procedures used (minority mutations at different frequencies can be revealed by ultradeep nucleotide sequencing, and they might have been subjected to negative selection at different intensities). 

How could one describe a quasispecies? In the beginning, viral quasispecies were assessed based on the ability of a viral population to grow in a particular environment or to compete with another population in cell culture. Unfortunately, HCV in vitro cell lines/cultures are not optimal; thus, there is no system for analysing its phenotypic characteristics, and the only available study systems are genetic systems.

The “consensus sequence” includes all the nucleotides most frequently observed in a viral population. It is used to analyse the changes that affect the global population. The variability in the consensus sequence is employed to describe viral evolution, but it does not provide information about the structure of quasispecies, and it might not be represented in the population as a real sequence. The consensus sequence can be obtained from direct sequencing of polymerase chain reaction (PCR) products, from the alignment of cloned sequences, or from ultradeep analysis. The term”haplotype” indicates a set of genomes with the same nucleotide genome.

The “master sequence” represents the most common sequence detected in the viral population at a specific time point, and it does not usually correspond to the consensus sequence. Generally, the master sequence represents the sequence with the highest fitness.

The term “complexity” refers to genetic information. Measurement of the quasispecies complexity involves quantifying the percentages of different variants (mutant frequency) and the polymorphisms among the variants. The most common parameters for determining the complexity of quasispecies are the following: (1) Mutation frequency is defined as the proportion of mutant nucleotides in a genome distribution relative to the consensus sequence; (2) Shannon entropy (SE) is defined as the proportion of different genomes or haplotypes in a mutant distribution; and (3) Hamming distance is defined as the number of amino acid differences between two sequences [35,36]. 

The Shannon entropy value is often normalized, log(N), where N is the number of clones, sequences, or haplotypes analyzed [37]. In this case, when all sequences are identical, the Shannon entropy value is ”0”, whereas “1” is the maximum value reached when all sequences are different. It can be calculated separately for synonymous and non-synonymous mutations [38]. Instead, to understand quasispecies dynamics and how they affect the intra-host adaptability of RNA viruses, the mutation rate should be distinguished from the mutation frequency [39]. In fact, the selection outcome includes a two-step process, mutation and selection, which can also be expressed as the mutation rate and mutation frequency, respectively. The mutation rate indicates the biochemical event of misincorporation (incorporation of one or more incorrect nucleotides) and generally refers to a specific nucleotide site. A mutation rate can be averaged over multiple sites and expressed as the number of substitutions per nucleotide copied.

The mutation frequency is the quantification of the proportion of mutant genomes in a viral genome population and is expressed as substitutions per nucleotide. The mutation frequency might refer to the proportion of a specific mutant type (often indicated as mutant frequency) or to any type of mutant (designated as mutation frequency). Mutation frequencies are influenced by the capacity of any mutant to produce progeny relative to the capacity of non-mutated classes or of other mutants produced in the same replicative ensemble. The mutation frequencies of RNA viruses range from 10^−3^ to 10^−5^ substitutions per nucleotide. Another distinction must be made between mutation rate and the rate of evolution, which indicates the “rate of mutation fixation”. The rate of evolution is generally calculated by comparing the consensus sequences of sequential viral isolates, and it includes a time factor so it is commonly expressed as substitutions per nucleotide per year. The rate of evolution estimated for HCV is in the range of 8 × 10^−4^ to 2 × 10^−3^ s/n/y [40,41].

Within host evolution, this can be represented by the dN/dS ratio (the ratio of non-synonymous mutations to synonymous mutations). Non-synonymous substitutions change the amino acid sequence of a protein. Synonymous mutations can be assumed to be neutral because they do not affect the amino acid sequence. Thus, a dN/dS ratio > 1 is indicative of positive selection on the viral population, and a dN/dS ratio < 1 indicates “negative selection” (or purifying selection), which occurs when non-synonymous mutations do not confer any advantage and are eliminated [42,43]. In HCV, the increase in non-synonymous mutations is often related to immune pressure [44,45,46]. Furthermore, an analysis of dN/dS can differ if one considers the whole protein, region, or single amino acid sites.

The term “virus fitness” was originally defined as the capacity of a virus to produce infectious progeny in a given environment and it is still widely used today [47].

The effective population size can also undergo a transient reduction in the number of viral genomes. This phenomenon is called a “bottleneck” and occurs in response to several factors, such as the immune response, limited availability of susceptible cells, or drug administration.

The increasing mutational load due to successive bottleneck events causes very low fitness achievement; however, there is a remarkable resistance to extinction and the genomes are rescued by compensatory mutations. 

In a longitudinal study carried out over 2 to 6 years with the pyrosequencing method, the authors demonstrated the correlation between genetic diversity and humoral selective pressure [48]. The genetic diversity was analysed in four different regions of HCV (5′UTR, core, HVR1, E2) during the acute phase of HCV infection. 5′UTR amplicons showed little diversity, both in HCV-monoinfected and in HIV/HCV-coinfected patients, at all of the time points analysed. In contrast, samples from the HVR1 and E2 regions exhibited a higher degree of variation. In the longitudinal analysis of the 5′UTR and core regions, the Shannon entropy values were low (<0.3), and they did not increase over time. In contrast, the Shannon index values for the envelope amplicons were significantly higher than those for the 5′UTR and core sequences (*p* < 0.02 for pairwise comparisons), and they increased over time (*p* < 0.05). In the HVR1 region, 124 different amplicons were identified vs. <20 in 5′UTR. Moreover, for 5′ UTR amplicons, almost no diversity was detected and there was no difference between monoinfected and coinfected patients. Early after infection, the viral population comprised only few OTUs (operational taxonomic units), both in the conserved and variable region, though viraemia was >50,000 IU/mL, suggesting that only few variants were transmitted.

High diversity levels were observed for the core and E2 (excluding HVR1) amplicons in both groups (monoinfected and coinfected), but the differences in the mean Shannon index values were not significant. Conversely, the HVR1 envelope region showed significantly greater diversity in the monoinfected subjects than in the coinfected individuals (*p* = 0.02616). These data could be explained by the impaired immune function in HIV patients diminishing the selective pressure on the HCV population such that it diversified to a lesser extent [48]. 

Bottleneck could be affected by host factors, such as IL-28B rs12979860 polymorphism [49,50]. In addition, higher values of synonymous Shannon entropy have been observed in subjects with IL-28B (IFNL3) rs12979860 CC genotype (vs non CC) across the near full-length of ORF in core, E2, NS2, NS3, NS4B, and NS5A protein coding segments [38].

## 3. Humoral Immune Response in HCV Infection

Humoral immune responses have long been believed to play a marginal role in the outcome of HCV infection [6]. Early studies measuring serum reactivity against HCV recombinant proteins or peptides demonstrated that although antibodies (Ab) specific for both structural and non-structural viral proteins were readily detected in the serum of chronically infected individuals, the antibody response was delayed in appearance and in relatively low titres during acute infection [51,52,53]. Nevertheless, initial studies in chimpanzees provided the first evidence that neutralizing antibodies (nAbs) targeting the HVR1 region of envelope glycoprotein 2 were raised during HCV infection and that they could protect chimpanzees against HCV challenge [54,55]. In humans, analysis of serum reactivity against the main HVR1 variant in a large group of patients who were infected by the same HCV isolate revealed that 43% of the resolved patients developed anti-HVR1 antibodies within the first 6 months after infection compared to 13% of the chronically evolving individuals, suggesting a contribution of neutralizing antibodies in virus clearance [56].

The development of infectious retroviral HCV pseudotypes bearing HCV glycoproteins has significantly advanced the study of nAb responses during HCV infection [57]. Early studies using HCV pseudoparticle (HCVpp) expressing heterologous envelope sequences demonstrated that the majority of chronically infected patients had high titres of cross-reactive nAbs. In contrast, these antibodies could be detected in only a minority of acutely infected individuals independent of viral clearance [58,59]. However, when individualized pseudoparticles were used to measure neutralization by autologous serum in a patient infected after blood transfusion, the development of nAbs against the autologous virus was detected 7 weeks after infection, coincidentally with acute viraemia and seroconversion [58]. Later, studies using autologous HCVpp to analyse the impact of neutralizing antibodies on the resolution of hepatitis C infection demonstrated a correlation between the presence of nAbs in the acute phase of the infection and both control of viraemia and spontaneous resolution [60,61]. In addition, a library of HCV pseudoparticles, which is representative of the most prevalent and incident subtypes of genotype 1 (GT1), was used to prospectively assess the anti-GT1 nAb responses during acute infection in at-risk persons. This study demonstrated that self-limiting infection was associated with early induction of broad nAb responses, which diminished after viraemia control. In contrast, in chronically infected individuals, nAb responses were delayed and progressively expanded, providing clear evidence of nAb responses in the control of acute HCV infection [62]. More recently the characterization of broadly neutralizing mAbs (bNAbs) from two persons who spontaneously cleared HCV infection demonstrated that they were similar to neutralizing antibodies from chronic infection in terms of clonality and epitope specificities, but they displayed a low number of somatic mutations [63]. Interestingly, a recent study demonstrated that polymorphisms outside the antibody binding epitopes in E1E2 confer resistance to neutralization by two of the most potent human bNabs, suggesting a novel mechanism by which HCV might persist [64].

The demonstration that the development and early responses of nAbs were correlated with both viremia control and spontaneous resolution of HCV infection led to an investigation of the impact of humoural immune responses on HCV envelope sequence evolution and the generation of neutralization escape. 

A study on envelope glycoprotein sequence evolution and serum neutralization carried out in eight acutely infected individuals demonstrated that antibodies neutralizing earlier sequence variants were detected at earlier time points than antibodies neutralizing later variants, indicating that pressure from nAbs effectively drives viral sequence evolution. The rapid evolution and greater variability of envelope genes with the rest of the HCV genome suggested that circulating viral quasispecies are modulated by ongoing immune pressure. The majority of substitutions occurred in HVR1 amplicons during acute infections, and selective pressure exerted by nAbs drives sequence evolution, clearing circulating viral variants in resolver patients or causing a replacement with escape variants when viremia persists. All resolvers possessed high-titer nAb responses that peaked at time of viral clearance, whereas all chronic progressors possessed low-titer or absent responses throught the acute phase [65].

Compelling evidence that viral escape from nAb responses continues after decades of chronic infection was obtained from a study of a chronic HCV patient monitored over a 26-year period. HCVpp expressing HCV glycoproteins circulating at different time points over 26 years were tested for their sensitivity to neutralization by autologous serum antibodies. Neutralization assays revealed that serum antibodies showed reduced neutralization of HCVpp bearing concurrent and later glycoproteins, although the antibodies efficiently neutralized HCVpp bearing glycoproteins circulating at earlier time points during infection [66]. The dominance of neutralization-resistant sequences in the viral population at each time point indicated that during chronic infection, the selective pressure exerted by humoral immune responses favoured the continuous generation of escape variants. Thus, these results indicated that the evolution of HCV envelope proteins is shaped by nAb pressure. Furthermore, genetic drift could also influence HCV evolution. It seems to be independent from the humoral response to HVR1 and could be essentially affected by replication errors and the replication cycles [67].

## 4. Role of CD8^+^ T Cells in HCV Evolution/Variability

Current estimations suggest that 20%–50% of the viral mutations observed over time in primary HCV infection are driven by CD8^+^ T cell responses [68]. 

These cells are believed to contribute to HCV clearance because, in humans, their appearance coincides with a rapid decrease in viraemia [69]. Similarly, in acutely infected chimpanzees, increases in CD8^+^ and IFN-γ mRNA expression in the liver coincided with a 90% to 99% decrease in viral titre [70]. In contrast, in chronically evolving acutely infected patients, HCV-specific CD8^+^ T cells progressively lose their ability to proliferate, to secrete IFN-γ, and to be cytotoxic [71]. In chronic infection, impairment of HCV-specific CD8^+^ T cells has been ascribed to the up-regulation of inhibitory molecules, such as programmed cell death (PD-1), cytotoxic T-lymphocyte-associated antigen 4 (CTLA-4), and T-cell immunoglobulin and mucin domain–containing molecule 3 (Tim-3). Blockade of these inhibitory receptors was able to restore the proliferation and effector function of CD8^+^ T cells in vitro [72,73,74,75,76].

The initial description that CD8^+^ T cells select for HCV escape mutations was obtained in chimpanzee studies [77,78]. Indeed, the analysis of HCV epitopes’ evolution in three chronically infected chimpanzees revealed the occurrence of mutations during the first weeks after infection, and these mutations persisted for years [78]. In humans, the first evidence that cellular immune pressures could be exerted during HCV infection was obtained by analysing viral evolution in an HLA-B8-restricted NS3 epitope targeted by dominant CD8 T cell responses in two acutely infected patients. In both individuals, multiple amino acid substitutions accumulated in the epitopic region, leading to variants that could not be efficiently recognized by CD8^+^ T cells [79]. Similarly, the study of two patients acutely infected from a single source revealed that chronic evolution of the disease was associated with the emergence of escape mutations in an epitope of the NS3 protein targeted by a vigorous CD8^+^ T cell response [80]. 

Furthermore, through the simultaneous comparison of HCV sequence evolution and comprehensive screening of T cell reactivity, Cox et al. demonstrated amino acid substitutions in at least one epitope targeted by CD8^+^ T cells from seven chronically evolving patients. In contrast, no substitution in T cell epitopes occurred in a subject who spontaneously cleared the infection [81]. Moreover, population-based studies have revealed that certain polymorphisms in viral proteins are associated with the expression of particular HLA class I molecules, providing additional evidence of HCV adaptation to CD8 T cell responses [82,83,84,85].

Later, important insights into the timing and emergence of escape variants in HCV acute infection have been obtained from studies of both viral evolution and host cellular immune responses in three HCV-infected individuals who were followed from pre-seroconversion until infection outcome. High resolution analysis of viral diversity by deep sequencing revealed that multiple novel variants evolved early in infection from the transmitted/founder (T/F) virus. In this phase of the infection, the rapid induction of virus-specific CD8^+^ T cells was associated with the elimination of the T/F virus, and the majority of variants were eliminated within 100 days’ post-infection. Thereafter, an increase in viral load was observed, which coincided with the occurrence of dominant immune escape variants, and the initiation of the chronic phase of the disease [86]. In addition, the likelihood of immune escape was significantly associated with the magnitude of the CD8^+^ T cell responses, indicating that adaptation of the virus to a new host is characterized by high and rapid variability in epitopes under CD8^+^ T cell immune pressure. 

Although the development of viral escape has been associated with a dramatic decrease in the frequency of circulating CD8^+^ T cells that target the escape epitopes, recent data have suggested that such CD8^+^ T cells retain the ability to exert selective pressure during chronic infection. Indeed, it has been demonstrated that pregnancy-induced impairment of HCV-specific CTL results in temporary reversion of HCV escape mutations and in an increase in HCV titres. After delivery, re-acquisition of escape mutations in one or more HLA class I epitopes coincided with the detection of CTL responses, indicating that CD8^+^ T cells targeting revertant epitopes retain in vivo function during chronicity [87]. The preservation of T cell function in CD8^+^ T cells targeting escape epitopes has been associated with the reduced expression of inhibitory coreceptors, suggesting that CTL targeting of escape epitopes is less functionally exhaustive than those targeting intact epitopes during chronicity [88,89]. 

Reversion to the wild-type sequence in the absence of CTL-mediated pressure driving the selection of escape mutants indicates a cost to viral replicative capacity [79,82]. Indeed, impairment of viral replication by CD8^+^ T cell escape mutations has been demonstrated in vitro [90,91]. In this case, to compensate for the detrimental effects of primary escape mutations on viral replication, additional substitutions within or outside the epitope are required. The occurrence of compensatory mutations has been demonstrated for immunodominant epitopes restricted by the HLA-A3, HLA-B27, and HLA-B57 molecules. These HLA alleles have been associated with spontaneous resolution of HCV infection and it has been proposed that the protection conferred by these HLA alleles is partly due to the restriction placed upon viral escape. In particular, the requirement of a specific cluster of mutations might delay the emergence of escape variants, providing sufficient time for the host immune system to successfully clear HCV [92,93,94]. Altogether, these data demonstrate that chronic evolution of HCV infection is associated with the accumulation of escape mutations in epitopes targeted by the acute phase CD8^+^ T cell response. In addition, the evidence that immune escape can coincide with mutations that reduce HCV replication indicates that the ability of the virus to mutate within CD8^+^ T cell epitopes could be limited by fitness constrains. 

## 5. CD4^+^ T Cell Responses in HCV Evolution/Variability 

There is a general consensus that cellular immune responses play major roles in determining the outcome of HCV infection [95]. The protective role of CD4^+^ T cells has been emphasized by multiple studies, which demonstrated that during acute HCV infection, the viral clearance is associated with the presence of vigorous and multispecific proliferative CD4^+^ T cells, while such responses were not detected in acute, persistently, or chronically infected individuals [95,96,97,98,99]. Moreover, in acutely symptomatic HCV-infected patients, the loss of virus-specific CD4^+^ T cell responses was immediately followed by recurrence of HCV RNA and chronic evolution of the disease [100]. However, a comprehensive evaluation of virus-specific CD4^+^ T cell responses using advanced CD4 T cell assays revealed that broadly directed HCV-specific T cell responses were universally detected in acutely infected HCV patients, suggesting that the lack of proliferative responses in patients with chronically evolving HCV does not always correspond to a lack of priming HCV T cell responses, but rather to functional defects in virus-primed CD4 T cells [101]. 

While it is generally recognized that CD8^+^ T cell-mediated selective pressure drives the accumulation of HCV escape mutations, the mutational escape in CD4^+^ T cell epitopes has been less frequently documented. Comprehensive analysis of the evolution of MHC class II-restricted viral epitopes in four chimpanzees persistently infected with HCV for more than 10 years demonstrated the emergence of an escape mutation in one CD4^+^ T cell epitope in only one of the studied animals. The observed mutation was detected as a minor variant three years after infection, and it became nearly fully fixed at six years. Furthermore, the evaluation of mutation rates in T cell epitopes in the four animals revealed that the frequency of non-synonymous mutations was higher in CD8^+^ T cell epitopes than in CD4^+^ T cell epitopes, suggesting that CD4^+^T cells play a minor role in driving viral evolution [102]. 

In accordance with these findings, quantitative analysis of sequence changes occurring throughout two NS3 epitopes targeted by CD4^+^ T cell responses in an acutely infected patient did not reveal the emergence of escape variants over a six month period [45]. Similarly, no epitope-inactivating mutations were observed by monitoring the sequence of two epitopes that were strongly immunodominant for intrahepatic CD4^+^ T cells in a patient with chronic HCV infection [103].

Despite this evidence, some data have indicated that CD4^+^ T cells can contribute to the evolution of the HCV E1/E2 glycoproteins. Indeed, the development of escape mutations in multiple CD4^+^ T cell epitopes located in the E1 and E2 proteins was demonstrated in a well-characterized HCV chronically infected patient [66]. In addition, viral variants that were not recognized by human HVR1 or NS3-specific CD4^+^ T cells have been reported [104,105]. More recently, evidence of HCV adaptation to CD4 T cell responses has been demonstrated at the population level by analyzing the host genotypes and autologous HCV sequences from 414 viremic subjects [106]. 

These data indicate that although escape mutations can occur in epitopes targeted by HCV-specific CD4^+^ T cells, they are less frequent compared to those in CD8^+^ T cell epitopes. 

## 6. IFN-Lambda Polymorphisms and HCV Infection 

Genetic polymorphism is defined as the inheritance of a trait controlled by a single genetic locus with two alleles, in which the least common allele has a frequency of about 1% or greater. Sources include single nucleotide polymorphisms (SNPs), sequence repeats, insertions, deletions, and recombination. SNPs (pronounced “snips”), are the most common type of genetic variation among people. Each SNP represents a difference in a single DNA building block [107].

Most SNPs have no effect on health or development. Some of these genetic differences, however, have proven to be very important in the study of human disease. Variants in the interferon lambda region have been associated with multiple HCV outcomes, including spontaneous clearance, treatment response, viral loads, and liver disease progression [49,108,109].

### 6.1. IFN Lambda Family

Humans have genes encoding four IFN-λ proteins (IFN-λ1 [IL-29], IFN-λ2 [IL-28A], IFN-λ3 [IL-28B], and IFN-λ4). Human IFN-λ1, IFN-λ2, and IFN-λ3 were identified in 2003 while IFN-λ4 was discovered in 2013 through RNA sequencing of primary human hepatocytes [110,111,112].

**Figure 2:**
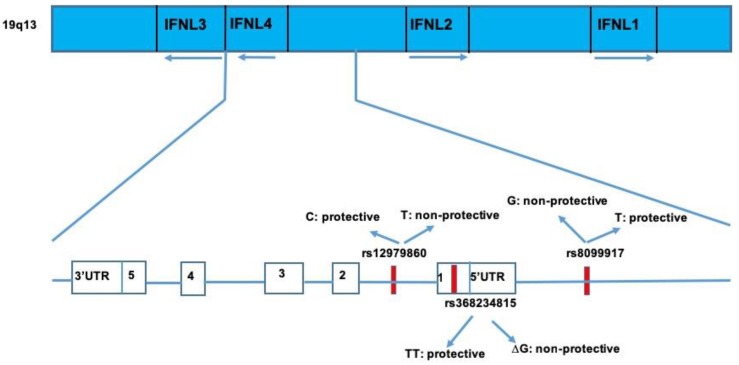
Representation of the interferon lamda 3-4 (IFNL) locus and IFN 1 and two loci in chromosome 19q13 with key single nucleotide polymorphisms (SNPs) involved in clearance of HCV infection.

Type III interferons belong to the IL-10 superfamily, but they are functionally related to type I interferons, which play a major role in antiviral immunity [113]. Their genes are located on chromosome 19 (Figure 2), and IL-28B is placed on the short arm of chromosome 19 (19q13.13) [113]. Type III interferons bind a unique receptor, which is composed of two subunits: IFN-λR1 chain (also indicated as IL-28RA), and interleukin-10 receptor 2 (IL-10R2), which is also included in the receptor complex for IL-10, IL-22, and IL-26. A study carried out with poxvirus demonstrated that a viral glycoprotein neutralized the activity of both type I and type III interferons with the exception of IFN-λ4, suggesting the existence of conserved epitopes between these two family members [114,115]. Similar to type I IFN, a signaling cascade, activated following IFN-λ binding to the receptor, leads to the activation of receptor-associated Janus Kinase 1 JAK1 and Tyrosine Kinase 2 Tyk2 kinases, which phosphorylate Tyr residues within the IFN-λR1 intracellular domain. These phosphotyrosine-based motifs serve as recruitment sites for the latent transcriptional factors of the signal transducer and activator of transcription STAT family, mainly STAT1 and STAT2 [116,117,118,119,120]. Phosphorylated STAT1 and STAT2 heterodimerize and interact with another transcription factor, IFN regulatory factor 9 (IRF9), leading to the formation of a transcription complex designated IFN-stimulated gene factor 3 (ISGF3). After that, ISGF3 binds to the IFN-specific response element (ISRE) that is commonly present in the promoter regions of hundreds of IFN-stimulated genes (ISGs), promoting their transcriptional expression [121]. However, under type I IFN stimulation, ISGs’ expression reaches a high level with a rapid decline, whereas IFN- III induces a slow, but prolonged expression of ISGs [121].

Despite the commonality of the proximal signaling events and downstream transcriptional responses between IFN-α/β and IFN-λ, the restricted expression of the IFN-λ receptor to a narrow spectrum of cell types and tissues makes the effects of IFN-λ most evident in epithelial cells [115]. 

### 6.2. IFNlambda3-IFN-Lambda 4 Polymorphisms and HCV Spontaneous Clearance 

Since 2009, several genome wide association studies (GWAS), aiming to identify host genes associated with the response to pegylated-interferon and ribavirin (PEG/RBV) treatment in HCV chronically infected patients, have been carried out in different world populations. All these studies showed the involvement of the variation in the IFNL3 (previously named IL-28B) gene region in spontaneous or treatment induced viral clearance [112,122,123,124,125,126].

Then, several studies investigated the impact of IL-28B genetic variation in the natural course of HCV infection. In 2009, genotyping of the rs12979860 variant in HCV cohorts comprising 388 individuals with spontaneous HCV clearance and 620 with persistent infection revealed that patients with the C/C genotype were three times more likely to clear the virus relative to patients with the C/T and T/T genotypes [49]. The allele frequency of the rs12979860 SNP differs between individuals with European or African ancestry, with the favourable rs12979860(C) variant predominating in the former population. This finding correlates with better clearance of HCV in European ancestry individuals. The analysis of the SNP, rs12979860, in a German cohort of women infected from a single GT1b source confirmed that patients with the C/T or T/T genotype had a lower chance of spontaneous clearance [127]. Similarly, a study examining genetic variation in the *IL28B* genomic region and the natural history of principally GT4 infections demonstrated that the rs12979860 C/C genotype is strongly associated with spontaneous clearance [128]. In addition, the analysis of nine prospective international cohorts evaluating outcomes following acute HCV revealed that genetic variation in the *IL-28B* gene independently predicted spontaneous clearance [129].

The first genome-wide association study assessing the influence of human genetic variation on the natural control of HCV demonstrated that the non-favorable rs8099917 GG genotype is a common genetic variant negatively associated with the spontaneous clearance of HCV infection [123]. These findings were corroborated by several studies describing *IL-28B* gene variance, including rs12979860 and/or rs8099917 being associated with high rates of spontaneous HCV clearances in Caucasians, African-Americans, and the Chinese population [130,131,132,133,134]. 

In 2013, a novel transcribed region located upstream of IFNL3 was identified. This region harbours a dinucleotide variant, ss469415590, that presents two alternative forms, TT and ΔG alleles. The ΔG variant generates the full-length protein designated as IFN-λ4, which shares only 29% homology at the amino acid level with the other three IFN-λs. The TT variant leads to a frameshift in exon 1 and disrupts the IFN-λ4 [112,135]. Surprisingly, IFN-λ4 expression is associated with decreased clearance of hepatitis C virus (HCV) in opposition to the lack of IFN-λ4 production that improves HCV clearance. The rs12979860 and rs8099917 IL-28B SNPs are located 367 bp downstream (intron 1) and 4 kb upstream of ss469415590, respectively. It has also been observed that the IFNL4-creating ss469415590 (rs 368234815) ΔG allele is in strong linkage disequilibrium (LD) with the unfavourable rs12979860-T allele; however, IFNL4-ΔG is associated with impaired HCV clearance more strongly than rs 12979860-T, especially in individuals from African ancestry in whom LD between the two alleles is weakest [136].

This result was confirmed in an analysis of 890 HIV/HCV coinfected woman showing that among blacks, HCV was more often cleared in subjects with genotype IFNL4-TT/TT in comparison to those with the TT/ΔG or ΔG/ΔG genotype [137]. Similarly, in a swiss cohort of 540 HCV infected patients, the IFNL4 TT/-ΔG was a better marker of spontaneous clearance compared with rs12979860 [126]. Also, in Brazilian HIV infected patients with the IFNL4 TT/TT genotype, the probability of a spontaneous clearance of HCV infection was 3.6 fold higher than for patients carrying the IFNL4 ΔG, while the CC genotype of the IFNL4 polymorphism had a 2.8 higher probability for spontaneous clearance [138]. However, other studies reported that IFNL3.rs12979860 and IFNL4.ss469415590 variants have comparable effects on spontaneous resolution of HCV in Egyptians, Iranian, and Chinese populations [139,140]. 

Linkage disequilibrium between IFNL4-ΔG, which creates IFN-λ4, and the (unfa-vorable) IFlL3 rs12979860-T allele is very high for Asians (r^2^ = 1.0) and Europeans (r^2^ > 0.9), which means that in these racial groups, IFNL4-ΔG and IFNL3 rs12979860-T almost always are inherited together [112]. For such highly linked variants, it can be difficult or impossible to determine which variant is more strongly associated with HCV infection outcome and, therefore, more likely to be causal. However, since among Africans, LD between IFNL4-ΔG and IFNL4 rs12979860-T is weaker, IFNL4-ΔG/TT is a plausible candidate as the causal variant undelying the observed genetic association with HCV clearance [136].

In addition, IFNL4 activity may influence the aminoacid polimorphisms in HCV. Indeed, a comprehensive host genome to viral genome analysis provided evidence that the host IFNL4 genotype influences viral NS5B polymorphisms as well as viral load through a mechanism dependent on a specific amino acid residue in the NS5A protein [141]. 

### 6.3. IFNlambda3-IFN-Lambda 4 Polymorphisms and HCV Therapy Outcome

Among SNPs identified in or near IL-28B region (i.e., rs12980275, rs8103142, rs8105790, rs1188222, rs8099917, rs12979860, etc.), only two of them, namely rs12979860 T/C and rs8099917 T/G, were considered relevant in antiviral treatment outcomes [124]. Both rs12979860 and rs8099917 SNPs frequencies vary among races. For example, the rs12979860 CC genotype is more common in Europeans and this could explain the higher percentage of SVR observed in European patients treated with PEG/RBV in comparison to Africans. A similar consideration could be done in Asians [142]. Other studies demonstrated that people harbouring the rs12979860 CC genotype had a 2 fold greater chance to achieve SVR than those with TT genotype; thus, the rs12979860 CC genotype is considered the strongest pretreatment predictor of the PEG/RBV response and its predictive power is independent on other pretreatment clinical factors, including the HCV genotype [143,144]. Furthermore, the rs12979860 CC genotype promotes a more rapid virological response (RVR) in pegylated and ribavirin therapy. Regarding rs8099917, the GG genotype has been identified as the strongest marker of treatment failure in Europeans and Japanese population [123,124].

The rs8099917 allele frequencies differ worldwide, and make their power in predicting an SVR weak in African–Americans [108].

The first DAAs to be cleared by the Food and Drug Administration (FDA) for use in the US, Telaprevir and Boceprevir, are NS3/NS4A serine protease inhibitors. The IL28B genotype significance was originally discovered in patients infected with HCV who were undergoing standard of care treatment (SOC) (pegylated interferon and ribavirin). The addition of a protease inhibitor to SOC, referred to as a triple therapy, has shown to significantly increase SVR rates up to 80% in HCV genotype 1 infected patients, but has called into question the relevance of IL28B polymorphism [145,146]. 

All studies on boceprevir/telaprevir showed an attenuated impact of IL-28B variants on the SVR, even though patients with a favourable rs12979869 CC genotype are more likely to be eligible for shorter treatment duration.

There is evidence that the favorable ‘‘responder” IFNL genotype is associated with higher hepatic inflammatory activity and an acceleration of fibrosis progression in CHC. It is noteworthy that multiple reports have shown that this effect is more predominant in subjects infected with HCV genotype 3 [147,148,149]. 

Few pediatric studies have explored the association between variations in the *IFNL3* gene and either spontaneous or treatment-induced clearance of HCV. The CC genotype of the rs12979860 SNP is associated with the spontaneous clearance of HCV in children independently of HCV genotype. Some pediatric studies have shown that both the rs12979860 CC genotype and the rs8099917 TT genotype are associated with the treatment-induced (IFN monotherapy and Pegylated-IFN-α and ribavirin association) clearance of HCV, while the rs12980275 SNP did not affect the virological response [150,151,152]. 

Another SNP of IL28B, rs12980275, was analyzed in chronic hepatitis C patients and different studies demonstrated it to be highly associated with SVR. A meta-analysis study showed that patients with the rs12980275 AA genotype, which are treated with pegylated-interferon and ribavirin, achieved more frequently SVR than patients with AG/GG genotypes in HCV infection genotype 1/4 [153]. 

A study showed that patients HIV-HCV coinfected with the rs12980275 A allele had higher fibrosis progression. In addition, considering the HCV genotype, patients infected with HCV-GT3 and with the rs12980275 A allele had higher odds of having values of alanine aminotransferase (ALT) ≥ 80 IU/L and reduced liver steatosis in HCV GT1 patients [154]. 

In predicting SVR in PEG/RBV treatment and possibly spontaneous HCV clearance in African–Americans, ss469415590 (IFNL4) has been proposed to be a better maker than rs12979860, whereas these variants seem to have similar informative capability in European-Americans [112].

This polymorphism appears to better predict the therapy failure in HIV/HCV coinfected patients in comparison to rs12979860 [155]. Moreover, IFNL4 polymorphism has been shown to be associated with RVR in patients under PEG/RBV therapy independently of the HCV genotype [156].

Rs469415590 (TT or ΔG) polymorphism shows a predictive role of SVR even in patients treated with new direct-acting antiviral agents (DAA) regimens without IFN [157].

More recently, the association of genetic variants of the IFNL4 (IL28B genotype) with HCV mutations that are associated with drug resistance (resistance associated variants, RAVs) has been investigated. The authors found that naturally occurring RAVs against NS3/4A protease inhibitors and non-nucleoside NS5B polymerase inhibitors were not significantly associated with the IFN-L4 genotype, whereas an association was found between Y93H NS5A RAV and IFN-L4 rs368234815 [158]. 

## 7. Conclusions

The data reported in this review shows how selective pressure exerted by the adaptive immune response influences within-host HCV evolutionary dynamics and adaptation during infection. 

HVR1 exhibited a higher rate of evolution most likely reflecting its role in evading humoral immunity.

Furthermore, several data demonstrated that the immune system preferentially selects viral mutations that escape T cell recognition. Large sequence studies of HCV, combined with the analysis of T cell function, have demonstrated that in humans, HCV evolution is predominantly driven by CD8^+^ T cells during acute viral infection; although functional constraints limit the degree of variation in the viral genomes. In contrast, a limited number of studies investigating the impact of virus-specific CD4^+^ T cells on HCV have indicated that escape mutations less frequently occur in HLA-class II restricted epitopes. These results highlight the importance of the adaptative immune system in driving viral evolution and serve as a map of the targets of T-cell immunity along the HCV, which can aid vaccine design. In addition, IFNL4 polymorphisms may significantly shape HCV genetic variability. Several studies have shown that favourable IFNL3-IFNL4 genotypes increase the chance of spontaneous resolution and interferon based treatment success. The strongest host genetic predictors of clearance are IFNL3-IFNL4 rs12979860, rs8099917, and rs368234815/ss469415590. This association suggests that IFNL3-IFNL4 typing might be useful for treatment decision making in acute infection, implying that patients with unfavourable IFNL3-IFNL4 genotypes could be prioritised for having early treatment, given their low likelihood of spontaneous clearance.
